# Determination of Suitable Macroporous Resins and Desorbents for Carnosol and Carnosic Acid from Deep Eutectic Solvent Sage (*Salvia officinalis*) Extract with Assessment of Antiradical and Antibacterial Activity

**DOI:** 10.3390/antiox10040556

**Published:** 2021-04-02

**Authors:** Martina Jakovljević Kovač, Valentina Pavić, Anastazija Huđ, Ines Cindrić, Maja Molnar

**Affiliations:** 1 Faculty of Food Technology Osijek, Josip Juraj Strossmayer University of Osijek, Franje Kuhača 18, 31000 Osijek, Croatia; mjakovljevic@ptfos.hr; 2 Department of Biology, Josip Juraj Strossmayer University of Osijek, Cara Hadrijana 8/A, 31000 Osijek, Croatia; vpavic@biologija.unios.hr (V.P.); anastazija.hud@biologija.unios.hr (A.H.); 3 Karlovac University of Applied Sciences, Trg J. J. Strossmayera 9, 47000 Karlovac, Croatia; ines.cindric@vuka.hr

**Keywords:** sage, resins, extraction, isolation, deep eutectic solvents, antiradical activity, antibacterial activity

## Abstract

In this study, for the first time, the adsorption/desorption characteristics of carnosic acid and carnosol from deep eutectic solvent extract of *Salvia officinalis* on five macroporous resins (HP20, XAD7HP, XAD16N, HP21, HP2MG) were evaluated. The high adsorption and medium desorption capacities of carnosic acid and carnosol as well as antibacterial and antiradical activity from the extract obtained with choline chloride:lactic acid (1:2) on XAD7HP resin indicated that resin was appropriate. To get the optimal separation process, the influence of factors such as adsorption/desorption time and volume of desorbent was further investigated. The results showed that the extract with high antiradical and antibacterial activity was obtained via adsorption and desorption on XAD7HP resin. The extraction efficiencies of the deep eutectic solvents (DESs) recycled once, twice, and thrice were 97.64% (±0.03%), 93.10% (±0.66%), and 88.94% (±1.15%), respectively, for carnosic acid, and 96.63% (±0.04%), 94.38% (±0.27%), and 91.19% (±0.36%), respectively, for carnosol, relative to the initial solvent efficiency. Based on that, this method is a promising basis for the large-scale preparation of extracts from *Salvia officinalis* with further application in the pharmaceutical or food industry, especially for maintaining the “green” character of the whole process to obtain the appropriate extract.

## 1. Introduction

The *Lamiaceae* family, with more than 236 genera and more than 7000 species, distributed worldwide, makes up the largest family within the *Lamiales* order. Numerous plants within the family have been recognized and cultivated for their flavor, fragrance, and positive health effects. Sage (*Salvia officinalis* L.), as a member of the *Lamiaceae* family, exhibits these properties, such as the pleasant aroma and positive health effects. These are the reasons for its use in culinary preparations and folk medicine, to treat various health conditions, such as inflammatory symptoms, respiratory problems, and mental and nervous disorders [1,2]. The complex composition of plants of the *Lamiaceae* family, including sage, includes bioactive components with an emphasis on terpenes (monoterpenes, diterpenes, triterpenes), flavonoids (lutein, apigenin, and quercetin) [3,4], and phenolic components (caffeic, vanillic, ferulic, and rosmarinic acids), which are highly bioactive with positive health impacts [5,6,7]. However, the antioxidant activity of sage is attributed to the presence of diterpenes such as carnosic acid, carnosol, and methyl carnosate [8,9], followed by flavonoids and other phenolic compounds [3]. The most important phenolic diterpene in sage is carnosic acid, from which, in the presence of oxygen and during harvesting and drying of leaves, an oxidative derivative, carnosol [10,11,12], is formed. Furthermore, during the extraction process, other diterpenes with lactone structure can be formed from diterpenes, such as rosmanol, epirosmanol, and 7-methyl-epirosmanol [13]. These components are essential as they have been shown to contribute more than 90% to the antioxidant activity of sage, in addition to exhibiting anticarcinogenic [5,14,15], antitumor, and anti-inflammatory properties [1].

Looking at the past few years, deep eutectic solvents (DESs) are increasingly used in various fields, especially in the extraction of phenolic components [16]. The reasons for the application of DESs in the extraction lie in the fact that they are easy to prepare and biodegradable with no or low toxicity, as well as the low cost of the starting components. Besides, numerous studies have shown that DESs can dissolve some components better than conventional organic solvents with an emphasis on lignocelluloses, thus achieving better mass transfer due to impaired cell structure [17]. Since DESs can be prepared from different starting materials as well as molar ratios, solvents are considered to be design solvents with tunable properties, achieving different extraction efficiencies for the desired components [18,19,20].

The main problem with the application of deep eutectic solvents in the extraction of phenolic components is the recovery of target compounds since deep eutectic solvents have negligible vapor pressure and generally high water miscibility [20,21]. Therefore, several methods have been proposed for the recovery of the desired components, such as chromatographic techniques, application of antisolvents, recrystallization, and back extraction [22,23]. 

In recent years, macroporous resins have been increasingly used to isolate and separate components from plants and plant extracts as they have proven to be effective, promising, and practical due to their unique properties (higher adsorption specificities and easier desorption) [24]. Compared to other methods, the advantages of this method include low cost, simple procedure, and high efficiency [25,26,27]. These advantages make macroporous resins successfully used in the separation and enrichment of different types of bioactive components, such as saponins, [25] paclitaxel, [26] isoflavone, [27] anthocyanins, [28], and levan [29] from natural resources. In this work, the recovery of the desired components was tested using five different macroporous resins by using the static adsorption experiment. 

Taking into account all the above, the objectives of this study were focused on (1) investigation on finding an appropriate macroporous resin for recovery of carnosic acid and carnosol, as well as (2) a suitable “green” desorbent. Afterwards, the influence of various parameters (time of adsorption and desorption as well as the volume of desorbent) on the (3) content of carnosic acid and carnosol in the sample analyzed by high performance liquid chromatography (HPLC) was investigated. Also, (4) the antiradical and antibacterial activity for the obtained sample was determined. Thereafter, the antiradical and antibacterial activity of the obtained samples was compared with the activity of the extract obtained by conventional solvents (5).

## 2. Materials and Methods 

### 2.1. Chemicals

A carnosic acid and carnosol standard as well as 2,2-Diphenyl-1-picrylhydrazyl (DPPH) were purchased from Sigma Chemical Co. (St. Louis, MO, USA). Other solvents were obtained from J.T. Baker (Radnor, PA, USA). All components for the preparation of eutectic solvents are commercially available from suppliers such as Sigma-Aldrich (St. Louis, MO, USA), Acros Organics (Waltham, MA, USA), and Gram mol (Zagreb, Croatia). Macroporous resins (XAD7, XAD4, XAD16) were purchased from Sigma-Aldrich (St. Louis, MO, USA) and HP21 and HP20 from Mitsubishi Chemical Holdings (Chiyoda City, Tokio, Japan). The characteristics of the macroporous resins, according to the manufacturer and other authors [30,31], are shown in Table 1.

### 2.2. Plant Material

Dried leaves of sage (*Salvia officinalis* L.) were used for further experiments. Moisture content (12.42% ± 0.06) and the particle size were determined according to the methods described by Jokić et al. (2018) [32]. Each measurement was performed in triplicate. Prior to extraction, the plant material was ground in a laboratory mill. All measurements were performed in triplicate.

### 2.3. Preparation of DES

After the initial DES screening described in our previous paper [33], DES containing choline chloride:lactic acid (1:2) was chosen to investigate the macroporous resin efficiency. A choline chloride-based deep eutectic solvent was prepared as described in our previous work [34], that is, by mixing choline chloride and lactic acid in a molar ratio of 1:2, after which the mixture was heated at 80 °C with constant stirring until a clear liquid was formed. After preparation of the DES, the solvent was diluted with water, in this case with 10% (*v/v*), cooled to room temperature, and used as such for the further extraction procedure.

### 2.4. Extraction of Bioactive Components from Sage (Salvia officinalis) Leaves

Ground dried sage leaves (*S. officinalis* L.) (50 mg) were mixed with 1 mL of choline chloride:lactic acid (1:2) DES containing 10% of ultrapure H_2_O (Millipore Simplicity 185, Darmstadt, Germany) (*v/v*), to reduce the viscosity and improve the diffusivity. The same extractions were also performed conventionally, using 50 mg of the plant and 1 mL of the solvent (water, 50%, 70% aqueous ethanol (*v/v*), ethanol, and methanol). 

DES samples were stirred at 1500 rpm in an aluminum block (Stuart SHB) on a magnetic stirrer under optimal conditions (70 °C and time of 68 min), determined in our previous work [33], to ensure the maximum amount of carnosic acid and carnosol in the obtained extract. To compare the results, as shown by Jakovljević et al. [33], sage extracts were prepared in the same manner (50 mg of plant with 1 mL of solvent) with conventional solvents (water, ethanol, aqueous ethanol solutions (30–70% (*v/v*)), and methanol). Immediately upon completion of the extraction, the samples were centrifuged for 15 min and then decanted. The supernatant liquid was diluted with methanol to prepare samples for HPLC and then filtered through a PTFE 0.45 μm filter.

### 2.5. Resin Pretreatment

The resins were prepared before isolation according to the manufacturer’s instructions. The desired amount of resin was transferred to a flask placed on a magnetic stirrer, and then a sufficient volume of ethanol was added to cover the resins and above 2.5–5 cm. The content was gently stirred for 1 min on a magnetic stirrer and then left at room temperature for 15 min. Then, ethanol was carefully decanted and replaced with Milli-Q water. The content was again mixed for 1 min and then left for 10 min. The resins thus prepared were filtered immediately before use and used for further procedure.

### 2.6. Static Adsorption and Desorption Properties of the Macroporous Resins

The recovery of the target components from DES extraction solution was carried out by static adsorption using different macroporous resins according to a modified method by Yang et al. [35]. An amount of 2.5 mL of DES extract was put into a 50 mL flask, and 1.0 g macroporous resin was added. The adsorption was performed at room temperature, approximately 25 °C and 200 rpm for 3 h. The macroporous resin was filtered out and then desorbed with 2.5 mL of different solvents (water, 50 and 70% ethanol solution, ethanol, and methanol) at approximately 25 °C and 200 rpm for 2 h. The carnosic acid and carnosol content in the DES extraction solution, the solution after adsorption, and the solution after desorption were determined separately. Accordingly, the adsorption capacity of macroporous resin *q* and adsorption yield *E*, as well as the desorption yield of solvents *D,* were calculated using the following equations:

(1) $$q=\frac{\left(\rho_{0}-\;\rho_{e}\right)\;\times \;V}{m}$$

(2) $$E=\;\frac{\rho_{0}-\rho_{e}}{\rho_{0}}\;\times \;100$$

(3) $$D=\;\frac{\rho_{d\;}x\;V}{Q}\;\times \;100$$

where *q* is the unit saturation adsorption capacity of resin (mg/g); *ρ_0_* is the concentration of carnosol and carnosic acid in extract (g/L); *ρ_e_* is the equilibrium concentration of carnosol and carnosic acid (g/L); *m* is the mass of resin (g); *V* is the volume of filtrate (L), *E* is adsorption yield (%), *D* is desorption yield, $\rho_{d}$ is the equilibrium concentration of carnosol and carnosic acid (g/L), and *Q* is the adsorption quantity of the resin (g) [35].

The process was performed in triplicate, and the results are expressed as the mean.

To examine the adsorption capacity of macroporous resins, the influence of adsorption and desorption times, as well as desorbent volume, was examined. The adsorption time was examined in the range of 60–360 min, while the desorption time was 60–1080 min. The volume of desorbent was in the range of 1–10 mL.

### 2.7. Recycling of DES and Macroporous Resins

After the process of adsorbing the extract onto the macroporous resin, the eutectic solvent was filtered and then evaporated to remove any residual water. The solvent was then prepared by adding 10% (*v/v*) water and reused for further extraction.

After the desorption process, the resins were treated as described in Section 2.5 and reused in the adsorption process of the components from the extract.

### 2.8. Chemical Characterization of the Obtained Extracts

HPLC analyses of carnosic acid and carnosol from sage leaves was performed on an Agilent 1260 Infinity II (Analytical Instruments, Santa Clara, CA, USA) with chromatographic separation on a ZORBAX Eclipse Plus C18 (Agilent, Santa Clara, CA, USA) column (100 mm × 4.6 mm, 5 µm). Separation of the analyzed compound was made with the method described in our previous paper [33].

### 2.9. Antiradical Activity

The antiradical activity of the extracts was examined using the DPPH (2,2-diphenyl-1-picrylhydrazyl) method according to the method previously described in detail [36]. Methanol DPPH solution (0.3 mM) was prepared daily and stored in the dark until analysis. The absorbance of the DPPH solution was measured before measuring the samples in the same way. Next, 1.2 mL of samples (concentration 250 µg mL^−1^) was mixed with 0.5 mL of DPPH solution and stored in the dark for 30 min. After a time (30 min), the absorbance was determined at 517 nm using a spectrophotometer (Helios γ; Thermo Spectronic, Cambridge, UK). For all samples, the measurement was performed in triplicate and compared with the control. The % DPPH inhibition was calculated according to the following formula:

(4) $$DPPH\;\left(\%\right)\;=\frac{\left(A_{DPPH}\;+\;A_{S}\right)-\;A_{P}}{A_{DPPH}}\times 100$$

where *A_DPPH_* is the absorbance of DPPH solution, *A_S_* is the absorbance of sample, and *A_P_* is the absorbance of blank. For selected samples that showed at a concentration of 250 ugmL^−1^ higher inhibition of DPPH radical, *EC_50_* was determined since it presents an easier tool for comparison of the results with the literature data where not only different methods but also different solvents were used [37]. The samples obtained by different volumes of ethanol on XAD7HP were used for calculating *EC_50_* values over the curve obtained from data on obtained relative scavenging capacity values and different concentrations of samples. 

### 2.10. Antibacterial Susceptibility Testing

#### 2.10.1. Microorganisms and Growth Conditions

Four investigated bacteria, *Bacillus subtilis*, *Staphylococcus aureus*, *Escherichia coli*, and *Pseudomonas aeruginosa*, were isolates from different clinical specimens acquired from the Department of Microbiology of the Public Health Institute of Osijek-Baranja County, Croatia. They were chosen as human pathogens reflecting gram-positive and gram-negative bacteria. Bacterial cultures were grown overnight in Muller Hinton Broth (MHB) (Fluka, BioChemica, Germany) under optimal conditions for each strain. The antibiotic gentamicin (BioChemica, Sauerlach, Germany) was dissolved in distilled water.

#### 2.10.2. Minimum Inhibitory Concentration (MIC)

MIC values were determined by a modified broth microdilution method [38] and defined as the lowest concentrations of the extracts which completely inhibited the growth of an individual strain. The method as described in our previous work [39,40] was used for testing using serially diluted extracts (200 to 6.25 μg mL^−1^). Each plate contained growth control (bacterial inoculum without extracts), background control (broth and ethanol), and antibacterial standard gentamycin. After the incubation for 24 h (37 °C, 5% CO_2_, and 50% humidity), a 3 h secondary incubation was carried out with triphenyl tetrazolium chloride. 

### 2.11. Statistical Data Processing

Two-way ANOVA analysis was performed to ascertain the impact of both resins and desorption solvents on quantitatively determined parameters. The quantitative data obtained were presented by mean values and standard deviations (±SD). Analysis of variance was followed by Tukey’s multiple comparison test. The correlation between studied parameters was calculated using Pearson’s correlation test. All tests were performed at a level of significance of α = 0.05. Statistical analysis was performed using Statistica 13. software (TIBCO Software Inc, Palo Alto, CA, USA, 2018).

## 3. Results and Discussion

**Figure 1:**
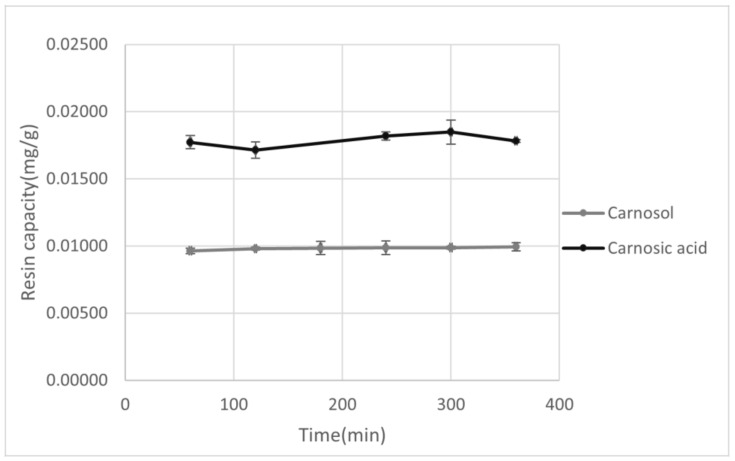
Graphical representation of the adsorption yield for XAD7HP of carnosol and carnosic acid as a function of adsorption time.

The adsorption and desorption capabilities of the macroporous resins depend on the target compounds and the adsorbent. Due to the chemical structures as well as different polarity, particle size, specific surface area, and pore diameter of different types of macroporous resins, it is hard to estimate their adsorption capacities for desired compounds. Therefore, in order to determine the best resin as well as a solvent for desorption of carnosic acid and carnosol for the first time, as shown in Figure 1, the experiment was performed with different resins and solvents, and at different process parameters including the time of adsorption and desorption as well as the volume of desorbent.

In the adsorption process, interactions between the adsorbent and the target compound, as well as with the solvent, are achieved [41]. According to Wang et al. [31], the process of the adsorption of phenolic compounds on the different macroporous resin is achieved via physical mechanisms between the adsorbent and the compound through van der Waals force or hydrogen bonding. 

Since polyphenols containing benzene rings and hydrogen groups may be of different polarity, five resins, including HP21, HP20, XAD16N, HP2-MG, and XAD7HP, were screened for the possibility of adsorption and desorption of carnosol and carnosic acid from the deep eutectic extract of *Salvia officinalis*. 

As can be seen from Table 2, all used macroporous resins exhibited similar adsorption behaviors and adsorption capacities for carnosol and carnosic acid. The HP21 and HP2MG resins showed proper adsorption capacity for carnosol of 0.0106 and 0.0109 mg/g, respectively. This was slightly higher than that of the other resins, XAD16N, XAD7HP, and HP20 (0.0104, 0.0102, 0.0101 mg/g, respectively). Among macroporous adsorption resins for carnosic acid, almost all resins displayed similar adsorption capacity (0.0256 and 0.0248 mg/g), and only HP2MG showed poorer performance compared to the other resins (0.0185), possibly due to the smaller surface area compared to the other resins. According to Li et al. [42] and Yang et al. [30], adsorption capacity is dependent on the chemical and physical properties of the resin, such as interaction forces, surface polarity, particle size, and surface area, as one of the most important factors. 

In this research, the surface area of HP21, HP20, XAD16N, HP2-MG, and XAD7HP was in the range of 470–800 m^2^/g, and since adsorption capacities for all resins are similar for carnosol and carnosic acid, it can be concluded that this range of surface area does not play a very significant role. On the other hand, studying the adsorption capacity, it can be seen that the highest percentage of adsorption of carnosol was achieved with HP20 and HP21 resins, whose surface area was 570–600 nm, while for carnosic acid, the high adsorption capacity was observed with HP20, XAD7HP, and HP21 resins, whose surface area was the range of 500–600 m^2^/g, while at the higher surface area (800 m^2^/g) and at the lower surface area (470 m^2^/g), smaller adsorption capacity was observed. Looking at desorption, XAD7HP and HP2MG were the most effective resins with a surface area in the range of 470–500 g^2^/m. 

In addition to the surface area, the pore size also affects the adsorption ability, since the solute needs to migrate through the pores to the adsorbing surface. Therefore, it is important that the pore size is large enough to retain the desired components, but if the pores are too large, large moles such as polysaccharides and proteins could be adsorbed, reducing the binding capacity for, in this case, carnosic acid and carnosol. Used macroporous resins have a pore radius in the range of 80–550 Å, and since adsorption capacities for all resins are similar for carnosol and carnosic acid, it can be concluded that this range of pore size does not play a very significant role. Nevertheless, it can be seen that the highest percentage of adsorption of carnosol was achieved with HP20 and HP21 resins, whose pore size is 80 and 260 Å, respectively, while for carnosic acid, the high adsorption capacity was observed with HP20, XAD7HP, and HP21 resins, whose pore size is 260, 550, and 80 Å, respectively. On the other hand, XAD7HP has a pore radius of about 550 Å, much higher than the other resins used in this paper. HP2-MG as the next resin with the best desorption has a pore size of 170 Å. Therefore, in this case, pore size cannot be related to desorption. 

The polarities of different macroporous resins depended primarily on their starting material. Generally speaking, according to the material, we can divide the resin into nonpolar macroporous resins, mostly composed of styrene and divinylbenzene polymers; moderately polar macroporous resins, mainly composed of polyacrylate polymers and multifunctional methacrylates used as crosslinking agents; the polar macroporous resin mainly contain sulfur, oxygen, and an amide group. According to Wang et al. [31], depending on the polarity of the components, the appropriate macroporous resin is selected in a way that the strongly polar components possessing benzene rings and hydrogen groups require moderately polar resin, while for weakly polar components, nonpolar resins were used, which is consistent with what we have shown in the paper. In contrast to the above groups in Wang et al. [31], in our paper, the emphasis is on hydroxy, carboxylic groups, and catechol moiety since the preferred components are carnosic acid and carnosol. In polystyrene adsorbents, additional adsorption properties include π-π interactions between the benzene ring and the components, while in polymethacrylic adsorbents, hydrogen bonds are formed between ester groups and components [30]. According to the adsorption results, it is possible that in both cases, although the bonds are different, strong enough bonds are formed between the resin groups and the groups of desired components. 

Since resins possess varying characteristics, they also exhibit different effects under the same conditions. According to Wang et al. [31], the adsorption and desorption process of macroporous resins to polyphenols from *Eucommia ulmoides Oliv*. was affected by many factors, such as those primarily mentioned, including solution properties. This is reflected in the example of polyamide resin, a common adsorbent widely used for separation as well as enrichment of bioactive components from Chinese plants where it was observed that, depending on the composition of the material used, the adsorption capacity is affected by the physical and chemical properties of the components as well [43].

Desorption of carnosic acid and carnosol from macroporous resin represents a competition of interactions between the intermolecular forces of adsorption and dissolution in the solvent. As can be seen from Table 2, the highest desorption is shown by the XAD7HP resin, in which the highest percentage of carnosic acid and carnosol was then desorbed into the selected solvent. On the other hand, in comparison to the adsorption, differences in the macroporous resins were observed in the desorption process. To be useful in the process of purification, adsorbed carnosic acid and carnosol should be easily desorbed under suitable conditions. Even though the ratio of desorption for all resins was not very high, the different resins’ desorption capacities can still be compared. Macroporous resin XAD7HP shows the highest desorption capacities, followed by HP2MG, while desorption capacities in the other resins are lower, about 20%, depending on the desorbent used. Since we have stated that the components bind to the resins with different bonds, so in polystyrene adsorbents, π-π interactions occur between the benzene ring and the components, and in the case of polymethacrylic adsorbents, hydrogen bonds occur between ester groups and components, we can conclude that binding strength affects desorption. According to the results, we can conclude that with macroporous resins containing methacrylate and acrylate, carnosic acid and carnosol are more easily released, indicating the weaker interaction between solute and the adsorbent material. Since the adsorption capacities of all resins were similar, the XAD7HP resin was selected based on the desorption yield.

The principle underlying the macroporous resin separation technique is the adsorption of the substance from the mixture onto the resin, which is washed with the selected solvent to remove the adsorbed components. Therefore, the eluent or desorbent is an important factor during the separation process, especially if the emphasis is on the use of GRAS (generally recognized as safe) solvents. The present study found that ethanol showed the highest efficiency for desorption of carnosic acid and carnosol, 47.47 and 47.08%, respectively, therefore, it was selected as the solvent for further testing. Not all used desorption solvents showed efficacy for desorption of carnosol and carnosic acid, so it is noticeable that in the sample obtained with water, there is almost no or a small desorption yield of carnosol and carnosic acid (11.63; 15.59%). The addition of ethanol and the preparation of aqueous solutions of ethanol (*v/v*) in the range of 50–70% ethanol showed better efficiency in the desorption of both components, so from the prepared aqueous solutions of ethanol, the highest efficiency for desorption of carnosic acid and carnosol was obtained with 70% ethanol (*v/v*) (34.78 and 42.04%). Nevertheless, absolute ethanol shows better efficiency in desorption of carnosic acid and carnosol compared to prepared aqueous ethanol solutions (47.47 and 47.08%). 

After selection of the resin and the appropriate solvent, adsorption capacity for XAD7HP resin was observed in the period of 60–360 min. As can be seen from Figure 1, with increasing absorption time, an increase in adsorption capacity was observed for both carnosol and carnosic acid, although with carnosic acid a maximum was observed at 300 min, after which there was a decrease in resin capacity. This does not have to be connected with the resin adsorption capacity itself but could be due to the possible decomposition of carnosic acid during prolonged exposure to air and light [44]. In the case of carnosol, a sudden increase in the adsorption yield with a longer absorption time was initially observed, but no significant difference was observed in the 250–300 min period, while an increase in resin adsorption was observed again in the period of 300–360 min. The reason for this increase may be the decomposition of carnosic acid into carnosol and other decomposition products, which explains both the decrease in the adsorption capacity of carnosic acid and the increase in the adsorption capacity of carnosol in the same time period. Due to this degradation, the appropriate adsorption time is in the range of 60–300 min. 

**Figure 2:**
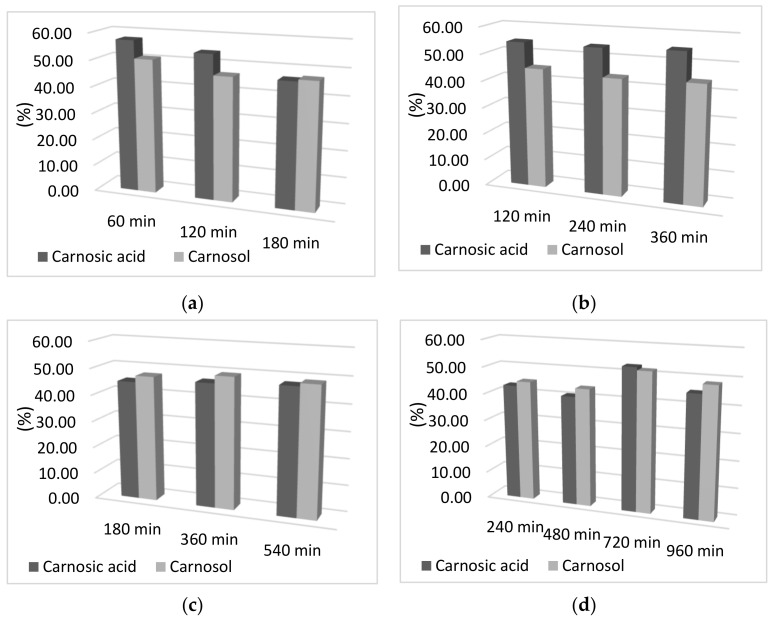

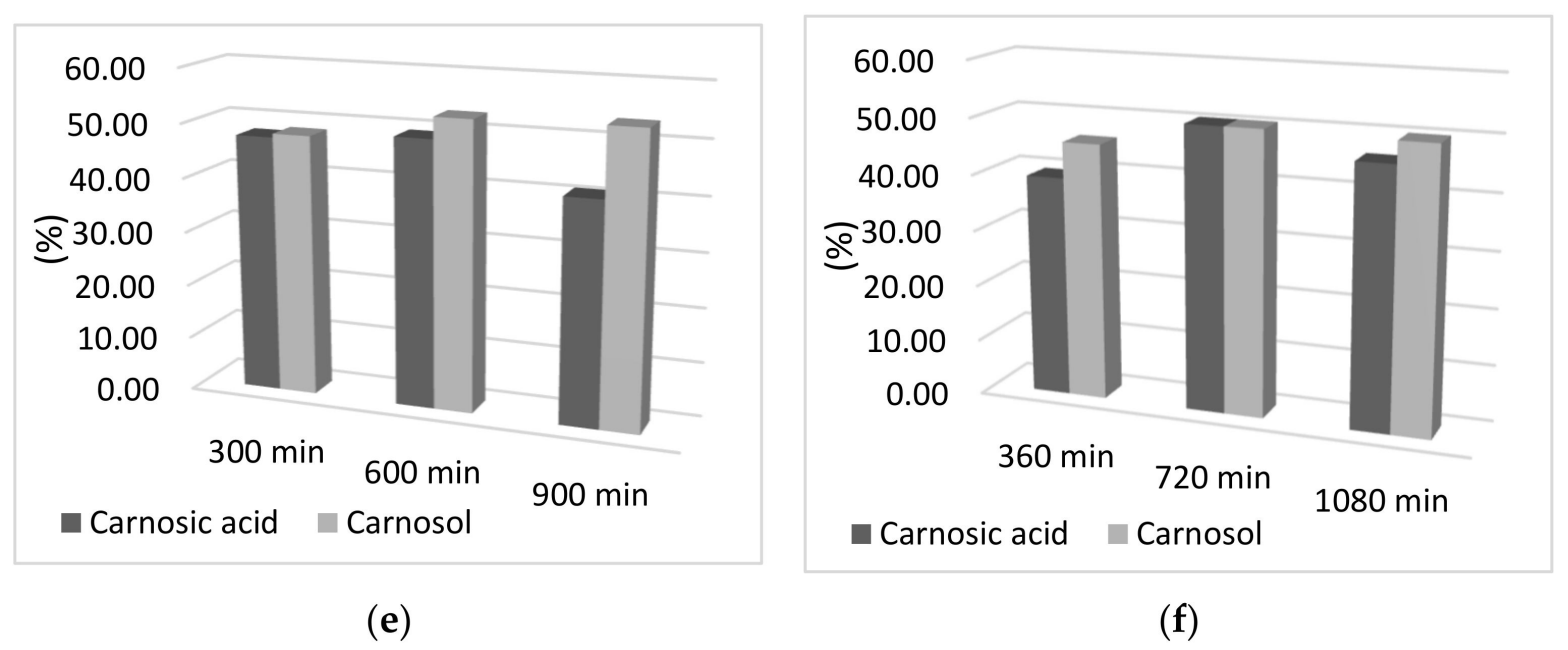
Graphical representation of the desorption yield of carnosol and carnosic acid as a function of desorption time; (**a**) 60 min adsorption; (**b**) 120 min adsorption; (**c**) 180 min adsorption; (**d**) 240 min adsorption; (**e**) 300 min of adsorption; (**f**) 360 min adsorption.

In addition to the influence of time on adsorption capacity, the influence of the adsorption and desorption times on the percentage of carnosic acid and carnosol in the final sample was examined. As can be seen from Figure 2, there was no difference in the percentage of carnosol and carnosic acid obtained by increasing the time of both adsorption and desorption. Moreover, a slight decrease in the percentage obtained for both components can be observed with increasing adsorption and desorption time, which may be due to the decomposition of the components due to a longer period of exposure to oxygen and light at room temperature. Therefore, the percentage of components is almost completely independent of the adsorption and desorption time, so the time of 60 min was selected for adsorption and desorption. Reducing the adsorption and desorption times not only reduces the exposure time of the sample to light, air, and room temperature, but also reduces energy consumption, which is important for maintaining the sustainability of the process.

**Figure 3:**
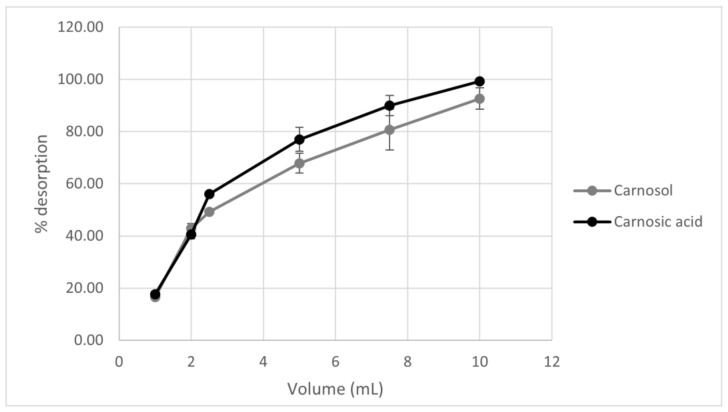
Graphical representation of the effect of desorbent volume on the desorption rate of carnosol and carnosic acid

Unlike desorption time, which does not affect the increase in carnosic acid and carnosol amount, the volume of solvent affects the amount of active components in the sample. In Figure 3, it can be seen that as the volume of ethanol as a desorbent increases, the desorption yield of both components also increases (Figure 3). Thus, desorbent volume is a key parameter for desorption with a volume of 10 mL of ethanol showing the highest desorption rate.

To our knowledge, this is the first work in which extraction was done with eutectic solvents, and isolation and purification with macroporous resins. In Patent US5256700A, the carnosic acid is adsorbed on an adsorbent solid material having an affinity for phenolic components, such as silica gel, aluminum oxide, polyamide, or polyvinylpyrrolidone. Thereafter, carnosic acid was desorbed using a polar solvent [45]. In the second case, carnosic acid was extracted using supercritical extraction followed by adsorption with different adsorbents. The fluid leaving the extractor was sent to the adsorption unit where the adsorbents were located and then to the separation vessel where the extract was recovered. This was followed by desorption process using 1–2% ethanol as a cosolvent to recover the components retained by the adsorbent. The adsorbents used were Mg silicate, silica gel, activated carbon, and Tonsil 180 FF, with activated carbon and Tonsil 180 FF proving to be the most effective [46].

**Figure 4:**
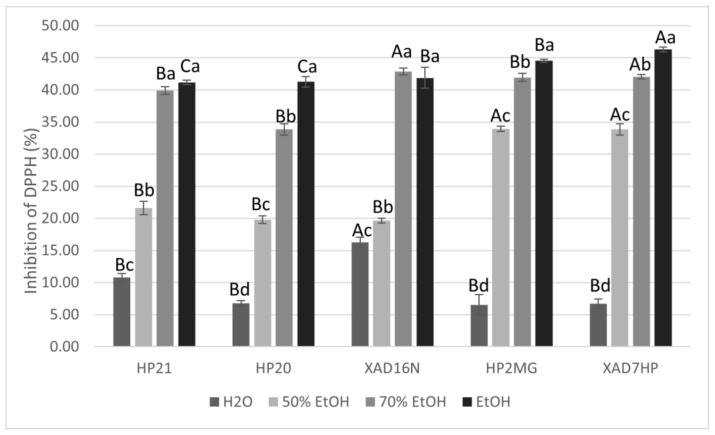
Graphic representation of % inhibition of DPPH with samples obtained with different resins and with different desorption solvents (ɣ = 250 µg/mL). Different uppercase letters indicate statistically significant differences (*p* < 0.05) between resins and different lowercase letters indicate statistically significant differences (*p* < 0.05) between desorption solvents.

For the prepared extracts with different resins and desorption solvents, the antiradical activity was examined by monitoring the percentage (%) of DPPH radical inhibition. As can be seen from Figure 4, the highest percentage of inhibition of DPPH radical was achieved via AMBERLITE XAD7HP resin using ethanol as the desorption solvent at a concentration of extracts of 250 µg/mL, which was statistically confirmed. Pearson’s correlation coefficient established an excellent positive correlation between DPPH inhibition and ethanol content (r = 0.91; *p* < 0.05), but it was found that the choice of macroporous adsorption resin showed only weak positive correlation with inhibition of DPPH radical (r = 0.30; *p* < 0.05). Pearson’s correlation coefficient established a good negative correlation of water content with DPPH inhibition (r = −0.85; *p* < 0.05). Samples were prepared as described in the methods, where the absorption on the resin lasted 3 h, while desorption into the solvents used lasted 2 h. Samples thus prepared show that at concentration of 250 µg/mL, approximately 50% inhibition of DPPH radicals was achieved when the desorption solvent was ethanol. Since the observed difference in the amount of carnosic acid and carnosol depended on the volume of the solvent, the EC_50_ was determined for these samples. In accordance with the increase in volume, not only desorption yield increases but also antiradical activity, which is observed by a lower EC_50_ value (Table 3). 

The antibacterial activity of sage leaf extracts was examined by microdilution test in Mueller–Hinton broth to determine their minimum inhibitory concentration (MIC). As shown in Table 4, the antibacterial activity of the extracts was equally effective in *E. coli* and *B. subtilis* and did not differ with respect to the macroporous resin or solvent used. Sage extracts showed different effects on *P. aeruginosa* with respect to adsorption macroporous resin and desorption solvent. DIAION HP20 and DIAION HP21 resins with 100% ethanol showed the most effective activity against *P. aeruginosa*. For other resins, 70% and 100% ethanol also proved to be effective. The results of antibacterial activity in *P. aeruginosa* depended on the ethanol concentration, which is in accordance with the results of [47] who found that antibacterial activity of the plant extracts varied depending on the level of ethanol used in the extraction. The least effective activity was against *S. aureus* strain. These differences in susceptibility between gram-positive and gram-negative bacteria can probably be attributed to structural and compositional differences in the membranes between the two groups. Due to the variation in the composition of active compounds, different plants may require different concentrations of ethanol to achieve maximum recovery of bioactive components. 

The results showed that the antibacterial activity of sage is affected by the solvent for desorption from macroporous resins. The percent of ethanol in the desorption solvent was found to enhance the antibacterial activity of sage extracts against *P. aeruginosa.* The resin type did not show a correlation with any parameter, except with MIC against *P. aeruginosa* where XAD7HP proved to be the most effective. It can be concluded that in this study, the choice of desorption solvent is highly important, in contrast to the choice of resin, which was not shown to be significant in this antibacterial susceptibility testing.

As shown in Table 5, the increase in 100% ethanol volume to 10 mL caused weaker extract antibacterial efficiency against all tested strains, which can probably be attributed to reaching the saturation point. Waszkowiak and Gliszczyńska-Świgło [48] showed that ethanol-to-water ratio of extraction solvent is an important factor affecting efficiency of phenolic compound extraction; an increase in ethanol volume in the tested solvents impacted negatively on extraction of most phenolic compounds. 

As shown in Table 6, minimum inhibitory concentrations of sage leaf extracts obtained with conventional solvents were much higher than DES extracts prepared with different resins and desorption solvents. 

**Figure 5:**
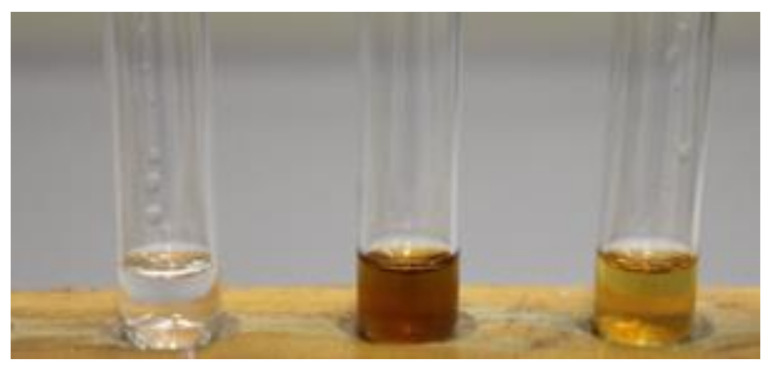
Representation of the pristine DES solution, DES solution containing the sage extracts, and recycled DES solution after resin adsorption (from left to right).

The extracts obtained this way contribute to the higher antibacterial activity compared to conventional extracts, while this method represents a possible way of recycling eutectic solvents and macroporous resins, which makes the process sustainable, with a minimum amount of waste. The use of macroporous resins for the adsorption of components from the extract obtained with DES ultimately yields a pure DES that can be reused for extraction (Figure 5). After adsorption, with adequate desorbent, the components are desorbed, and thus macroporous resin is recycled. The above was examined, and the results are given in Table 7. The extraction efficiencies of the DESs recycled once, twice, and thrice were 97.64% (±0.03%), 93.10% (±0.66%), and 88.94% (±1.15%), respectively, for carnosic acid and 96.63% (±0.04%), 94.38% (±0.27%), and 91.19% (±0.36%), respectively, for carnosol, relative to the initial solvent efficiency. These results show that DES can be recycled at least three times using macroporous resins to achieve a reasonably high level of carnosol and carnosic acid yields. With repeated application of macroporous resin, a decrease in the possibility of adsorption and desorption in three cycles is observed, although the values are still relatively high, which makes macroporous resins an appropriate adsorbent for the desired components and a means of recycling DESs.

## 4. Conclusions

In this study, for the first time, the process of static adsorption and desorption of carnosic acid and carnosol with five different macroporous resins from *Salvia officinalis* L. deep eutectic solvent extract was successfully achieved. Based on the static isolation results, XAD7HP was selected as a suitable resin and ethanol as a suitable desorbent for carnosic acid and carnosol enrichment, owing to its higher adsorption/desorption capacity. The most effective resin (XAD7HP) was successfully applied to obtain an extract with high antioxidant and antibacterial activity. According to the results, it is observed that the extracts obtained using deep eutectic solvents and then macroporous resins show much better antibacterial activity compared to classical extraction methods, which shows the effectiveness of this method of extraction and isolation and the possibility of further wide application in the food and pharmaceutical industries. Therefore, it can be concluded that the developed DES combined with macroporous resin enrichment established in this study presents an alternative method for green and efficient extraction and enrichment of carnosol and carnosic acid from the deep eutectic extract of *Salvia officinalis* without using toxic solvents. 

## Figures and Tables

**Table 1:** Physical and chemical properties of the macroporous resins.

Trade Name	Particle Size (μm)	Polarity	Pore Radius (Å)	Surface Area (m^2^/g)	Material
HP20	>250	Weakly polar	260	600	Polystyrene
HP21	250	Nonpolar	80	570	Polystyrene
HP2MG	>350	Moderately polar	170	470	Methacrylate
XAD16N	700	Nonpolar	150	800	Polystyrene
XAD7HP	500	Strongly polar	550	500	Acrylate

**Table 2:** Adsorption capacity, adsorption ratio of resins, and desorption ratio in different solvents.

Trade Name	Adsorption Capacity (mg/g)		Adsorption Yield (%)		Desorbent	Desorption Yield (%)	
	Carnosol	Carnosic acid	Carnosol	Carnosic acid		Carnosol	Carnosic acid
HP20	0.0101	0.0256	95.09 ± 3.75	100.00 ± 0.00	H_2_O	-	14.57 ± 0.09
					50% EtOH	12.65 ± 0.02	14.39 ± 0.01
					70% EtOH	15.42 ± 0.06	16.64 ± 0.11
					EtOH	24.15 ± 0.09	20.63 ± 0.17
XAD7HP	0.0102	0.0256	91.75 ± 2.72	100.00 ± 0.00	H_2_O	-	-
					50% EtOH	20.17 ± 0.06	20.74 ± 0.18
					70% EtOH	42.04 ± 0.19	34.78 ± 0.22
					EtOH	47.08 ± 0.25	47.47 ± 0.05
XAD16N	0.0104	0.0248	93.58 ± 2.42	97.06 ± 6.57	H_2_O	-	-
					50% EtOH	10.94 ± 0.05	14.96 ± 0.20
					70% EtOH	18.26 ± 0.09	23.37 ± 0.12
					EtOH	29.62 ± 0.15	28.69 ± 0.11
HP21	0.0106	0.0256	95.50 ± 3.70	100.00 ± 0.00	H_2_O	8.61 ± 0.01	-
					50% EtOH	9.51 ± 0.31	-
					70% EtOH	15.54 ± 0.05	16.61 ± 0.13
					EtOH	28.72 ± 0.07	20.85 ± 0.12
HP2MG	0.0109	0.0185	90.96 ± 2.74	87.52 ± 3.26	H_2_O	11.63 ± 0.22	15.59 ± 0.26
					50% EtOH	18.04 ± 0.02	16.55 ± 0.23
					70% EtOH	29.67 ± 0.05	27.38 ± 0.07
					EtOH	43.90 ± 0.24	40.32 ± 0.01

**Table 3:** Display of EC_50_ values for extracts obtained with AMBERLITE XAD7HP resin with different volumes of ethanol.

Sample	Absorption Time (min)	Desorption Time (min)	Volume of Ethanol (mL)	EC_50_ (μg mL^−1^)
XAD7HP	60	60	1	379.50 ± 13.62
			2	314.87 ± 6.25
			2.5	266.12 ± 5.62
			5	237.26 ± 3.57
			7.5	233.19 ± 1.81
			10	211.76 ± 2.29

**Table 4:** Comparison of minimum inhibitory concentrations (MIC) of DES sage leaf extracts against *Escherichia coli*, *Pseudomonas aeruginosa, Bacillus subtilis*, and *Staphylococcus aureus* (μg mL^−1^).

Trade Name	Desorbent	Desorption Time (h)	Desorbent Volume (mL)	Minimum Inhibitory Concentration(μg mL^−1^)			
				*S. aureus*	*B. subtilis*	*P. aeruginosa*	*E. coli*
Diaion HP20	H_2_O	2	2.5	50	25	25	25
	50% EtOH			50	25	25	25
	70% EtOH			50	25	25	25
	EtOH			50	25	13	25
XAD7HP	H_2_O	2	2.5	50	25	13	25
	50% EtOH			50	25	13	25
	70% EtOH			50	25	13	25
	EtOH			50	25	13	25
XAD16N	H_2_O	2	2.5	50	25	25	25
	50% EtOH			50	25	25	25
	70% EtOH			50	25	13	25
	EtOH			50	25	13	25
HP21	H_2_O	2	2.5	50	25	25	25
	50% EtOH			50	25	25	25
	70% EtOH			50	25	25	25
	EtOH			50	25	13	25
HP2MG	70% EtOH	2	2.5	50	25	13	25
	EtOH			50	25	13	25
Gentamicin				0.98	0.98	1.95	3.91

**Table 5:** Comparison of minimum inhibitory concentrations (MIC) of DES sage leaf extracts obtained with AMBERLITE XAD7HP resin with different volumes of ethanol against *Escherichia coli*, *Pseudomonas aeruginosa, Bacillus subtilis*, and *Staphylococcus aureus* (μg mL^−1^).

Trade Name	Desorbent	Desorption Time (h)	Desorbent Volume (mL)	Minimum Inhibitory Concentration(μg mL^−1^)			
				*S. aureus*	*B. subtilis*	*P. aeruginosa*	*E. coli*
XAD7HP	EtOH	1	1	50	25	13	25
			2	50	25	13	25
			2.5	50	25	13	25
			5	50	25	13	25
			7.5	50	25	13	25
			10	100	50	25	100
Gentamicin				0.98	0.98	1.95	3.91

**Table 6:** Comparison of minimum inhibitory concentrations (MIC) of sage leaf extracts obtained with conventional solvents against *Escherichia coli*, *Pseudomonas aeruginosa, Bacillus subtilis*, and *Staphylococcus aureus* (μg mL^−1^).

Solvent	Time (min)	Temperature (°C)	Minimum Inhibitory Concentration(μg mL^−1^)			
			*S. aureus*	*B. subtilis*	*P. aeruginosa*	*E. coli*
H_2_O	90	30	938	938	938	938
30% EtOH			469	469	469	234
50% EtOH			469	469	469	234
70% EtOH			469	469	469	234
H_2_O	90	50	938	938	938	938
30% EtOH			469	469	469	234
50% EtOH			469	469	469	234
70% EtOH			469	469	469	234
H_2_O	90	70	938	938	938	938
30% EtOH			469	469	469	234
50% EtOH			469	469	469	234
70% EtOH			469	469	469	234
DES	90	70	59	59	59	29

**Table 7:** Comparison of extraction efficiency, as well as adsorption and desorption yield, when reusing purified DES and recycled resin (60 min adsorption/desorption time).

Sample	Number of Cycles	Extraction Efficiency(%)		Adsorption Yield (%)		Desorption Yield (%)	
		Carnosic acid	Carnosol	Carnosic acid	Carnosol	Carnosic acid	Carnosol
XAD7HP	1	97.64 ± 0.03	96.63 ± 0.04	91.79 ± 0.65	94.28 ± 0.06	95.09 ± 1.22	92.51 ± 1.71
	2	93.10 ± 0.66	94.38 ± 0.27	83.07 ± 0.53	86.20 ± 0.48	89.38 ± 0.63	89.38 ± 0.88
	3	88.94 ± 1.15	91.19 ± 0.36	77.28 ± 1.42	78.23 ± 0.49	79.49 ± 1.04	84.94 ± 0.36

## Data Availability

The data presented in this study are available from the authors.
